# Monitoring the Emergence of SARS-CoV-2 VOCs in Wastewater and Clinical Samples—A One-Year Study in Santiago de Compostela (Spain)

**DOI:** 10.3390/v17040489

**Published:** 2025-03-28

**Authors:** Marta Lois, David Polo, María Luisa Pérez del Molino, Amparo Coira, Antonio Aguilera, Jesús L. Romalde

**Affiliations:** 1 CRETUS, Departament de Microbiología y Parasitología, CIBUS-Faculty de Biología, University de Santiago de Compostela, 15782 Santiago de Compostela, Spain; marta.lois.alvedro@usc.es (M.L.); david.polo.montaero@usc.es (D.P.); 2 Servicio de Microbiología, Hospital Clínico University de Santiago, 15706 Santiago de Compostela, Spain; maria.luisa.perez.del.molino.bernal@sergas.es (M.L.P.d.M.); maria.amparo.coira.nieto@sergas.es (A.C.); antonio.aguilera.guirao@sergas.es (A.A.)

**Keywords:** wastewater-based epidemiology, COVID-19, SARS-CoV-2, VOCs, duplex RT-qPCR

## Abstract

Wastewater surveillance has become a valuable tool to monitor the emergence of SARS-CoV-2 variants of concern (VOCs) at the community level. In this study, we aimed to evaluate the presence of Alpha (B.1.1.7), Beta (B.1.351), Delta (B.1617.2), and Omicron (B.1.1.529) VOCs in samples from the inlet of a wastewater treatment plant (WWTP) as well as from two different sewer interceptors (SI-1 and SI-2) from the urban sewage system in Santiago de Compostela (Galicia, NW of Spain) throughout 2021 and January 2022. For this purpose, detection and quantification of the four VOCs was performed using four duplex SARS-CoV-2 allelic discrimination RT-qPCR assays, targeting the S-gene. An N1 RT-qPCR gene assay was used as a reference for the presence of SARS-CoV-2 RNA in wastewater samples. All VOCs were detected in wastewater samples. Alpha, Beta, Delta, and Omicron VOCs were detected in 45.7%, 7.5%, 66.7%, and 72.7% of all samples, respectively. Alpha VOC was dominant during the first part of the study, whereas Delta and Omicron detection peaks were observed in May–June and December 2021, respectively. Some differences were observed among the results obtained for the two city sectors studied, which could be explained by the differences in the characteristics of the population between them. Wastewater-based epidemiology allowed us to track the early circulation and emergence of SARS-CoV-2 variants at a local level, and our results are temporally concordant with clinical data and epidemiological findings reported by the health authorities.

## 1. Introduction

The coronavirus disease (COVID-19), caused by severe acute respiratory syndrome coronavirus 2 (SARS-CoV-2), has spread worldwide since the first cases were reported in late December 2019, posing a serious risk to global public health and resulting in a noteworthy socio-economic impact. From the beginning of the pandemic, the detection of SARS-CoV-2 in the feces of infected patients and in wastewater has led to the possibility of the use of wastewater surveillance for epidemiological studies [1]. Wastewater-based epidemiology (WBE), initially developed to estimate illicit drug consumption in a community, is a comprehensive, cost-effective, and rapid technique that can provide essential information on a population’s health and behavior within a given wastewater system [2]. WBE also became a helpful public health tool for monitoring and managing COVID-19 disease at the population level [1,3,4]. Monitoring the presence in wastewater of SARS-CoV-2 RNA shed by people infected both with and without symptoms allows the detection and quantification of viral genetic material and serves as an early prediction that COVID-19 is spreading in a community [1,3]. In the last years, it has also been widely used to monitor a variety of viral pathogens, such as Poliovirus, Enteroviruses, Mpox Virus, Hepatitis A and E viruses, Noroviruses, Sapoviruses, and Rotaviruses, among others [5,6].

Over the course of the pandemic and due to the high mutation rate of SARS-CoV-2, new variants of the virus emerged and rapidly spread all over the world. Some of them had the potential to evade host immune responses, and were characterized by enhanced transmissibility and disease severity, and reduced effectiveness of vaccines with high re-infection rates [7,8,9,10]. These variants were designated by the WHO as variants of concern (VOCs) based on the risk posed to global public health (https://www.who.int/en/activities/tracking-SARS-CoV-2-variants/; accessed on 15 January 2025). Initially, the WHO classified five variants as VOCs: Alpha (Pango lineage B.1.1.7), Beta (lineage B.1.351), Gamma (lineage P.1), Delta (lineage B.1.617.2), and Omicron (lineage B.1.1.529) [11]. Alpha was first identified in the United Kingdom in September 2020 and became the predominant SARS-CoV-2 variant worldwide in early January 2021. Beta was first detected in October 2020 in South Africa. Delta was first detected in India in May 2021 and became the predominant variant worldwide in mid-July. Finally, Omicron was first detected in November 2021 in Botswana and South Africa and became the dominant variant worldwide in February 2022. Afterwards, multiple Omicron subvariants emerged and were classified as VUMs (variants under monitoring) and VOIs (variants of interest). At the time of writing, JN.1 is the dominant VOI circulating worldwide, and several VUMs are being tracked, namely JN.1.1, KP.2, KP.3, KP.3.1.1, LB.1, and XEC [12].

Tracking the emergence and propagation of VOCs in a community through wastewater-based epidemiology has become essential to fight against the COVID-19 pandemic [4,7,8]. WBE can provide a comprehensive view of most SARS-CoV-2 variants spreading in a monitored population during the sample collection period, identifying the dominant variant(s) and offering crucial insights into the introduction, transmission dynamics, and epidemiology of variants of concern (VOC) within the community [13,14,15,16,17,18,19,20,21,22,23,24,25,26,27,28].

The most common methods used to monitor the presence of SARS-CoV-2 in wastewater are genomic sequencing and the RT-qPCR (Reverse Transcription-Quantitative Polymerase Chain Reaction) or RT-dPCR (Reverse Transcription-Digital PCR). Genomic sequencing of SARS-CoV-2 in wastewater can monitor the circulation of different VOCs and their geographical distribution but also detect new mutations from different lineages [14,16,20,21,22,25], occasionally before their discovery in clinical samples [29]. Using this technique, increases in the proportion of VOCs were observed earlier than clinical reporting in the respective regions [19,21]. However, sequencing is longer than RT-qPCR-based assays and involves costly infrastructures, requiring arduous bioinformatics analysis and expertise [23]. In this way, RT-PCR-based methods can rapidly detect and quantify variant-specific mutations of the SARS-CoV-2 genome even if VOCs are present at low concentrations and prior (days or weeks) to their identification by local clinical sequencing [13,15,17,18,19,20,22,23]. RT-qPCR and RT-ddPCR are often designed as duplex or multiplex to detect a signature mutation of a particular variant, thus giving an estimation of VOC percentages among other simultaneously occurring variants [23,27]. By simultaneously detecting multiple mutations or variants in a single reaction, these assays offer a rapid, efficient, and high-throughput method for quantifying and differentiating variants within a population.

In the present study, four different duplex quantitative real-time RT-qPCR assays were employed to detect Alpha, Beta, Delta, and Omicron variants in a wastewater treatment plant (WWTP) from Santiago de Compostela (A Coruña, Galicia, Spain), as well as two sewer interceptors (SI-1 and SI-2) collecting sewage from two areas in the city with completely diverse population characteristics. The presence and abundance of the different VOCs in wastewater samples were then compared to the results found in clinical samples.

## 2. Materials and Methods

### 2.1. The City

Santiago de Compostela (NW Spain) is not only the capital of the Autonomous Community of Galicia, but also a UNESCO World Heritage City thanks to its monumental beauty and extraordinary conservation and also for being the final destination of the Way of St. James, a thousand-year-old pilgrim route. With a stable population of approximately 100,000 inhabitants, it receives more than 800,000 national and international visitors every year. The old town (historic center) attracts most visitors and is home to not only the cathedral and the main monuments of the city, but also a large number of hospitality establishments, including restaurants, hotels, tourist apartments, etc. This part of the city is surrounded by modern neighborhoods with more stable populations of local inhabitants, which may also present some mobility as they accommodate some of the students from the University of Santiago de Compostela.

At the beginning of the year 2021, Spain was under a state of alarm (approved by the Government on 25 October 2020) that was extended until May, including limitations of social gathering, human mobility, nighttime curfews, restrictions regarding bar and restaurant opening times, etc.). From this month, and for the rest of the studied period, the city received a total of 178,913 visitors, 56,785 being foreign travelers from more than 100 countries all over the world (see Supplementary Figure S1) (https://oficinadelperegrino.com/en/statistics-2/; accessed on 18 December 2024).

### 2.2. Wastewater Sampling

**Figure 1:**
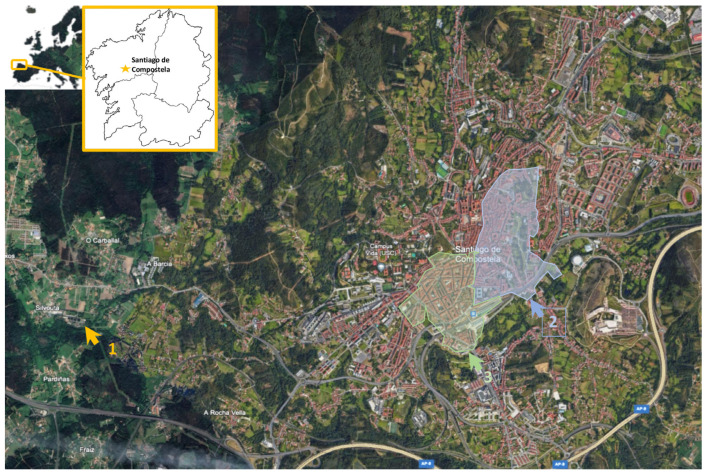
Geographic location of the study’s sampling locations (arrows) at Santiago de Compostela, A Coruña, Galicia, NW Spain. 1, WWTP; 2, SI-1; 3, SI-2. Source of color picture: Google Earth (data accession 17 March 2024).

Sewage samples were collected from early January 2021 to late January 2022 (56 weeks; Supplementary Tables S1–S3) from the inlet of Santiago de Compostela WWTP (*n* = 127), the only WWTP in the municipality that has a treatment capacity of 54,560 m^3^/day and an equivalent population of 184,000 inhabitants. Samples were also collected from two sewers interceptors, SI-1 (*n* = 52) and SI-2 (*n* = 52), in the city (Figure 1). SI-1 collect wastewater from the old town (an estimated population of 10,200 inhabitants), whereas SI-2 serves a neighborhood of local inhabitants with a stable population (an estimated populationof 6500 inhabitants).

Three (weeks 1 to 15) or two (weeks 16 to 56) times per week, 500 mL of each 24 h composite sample were collected early in the morning (8–9 am) and transported with ice blocks to the laboratory by the municipal water company, taking approximately half an hour. Samples were kept refrigerated at 4 °C upon arrival at the laboratory and processed within 24 h.

### 2.3. Sample Concentration and Viral Nucleic Acid Extraction

Wastewater samples (200 mL) were concentrated using the aluminum hydroxide adsorption-precipitation method as previously described [13,30]. Briefly, 2 mL of a 4% AlCl_3_ solution were added to each wastewater sample, followed by agitation for 15 min at 150 rpm. After agitation, the samples were centrifuged at 1700× *g*–1800× *g* for 20 min. The pellets were then resuspended in 10 mL of a 3% meat extract solution, mixed for 10 min at 200 rpm, and centrifuged again at 1900× *g*–2000× *g* for 30 min. Finally, the pellets were resuspended in 1–2 mL of phosphate-buffered saline (PBS). All wastewater samples were spiked with 20 µL (final concentration 2.5 × 10^5^ genomic copies (GC)/mL) of murine hepatitis virus strain A59 (MHV-A59), an animal coronavirus used as a viral process control [31].

Viral RNA from the concentrates was extracted using the Nucleospin^®^ RNA/DNA Virus Kit (Macherey-Nagel GmbH & Co., Düren, Germany), following the manufacturer’s instructions. Then, 150 µL of the concentrated sample was mixed with 25 µL of Plant RNA Isolation Aid (Thermo Fisher Scientific, Vilnius, Lithuania) and 600 µL of lysis buffer from the NucleoSpin Virus kit. Viral RNA was eluted in 50 μL of RNAse-free dH_2_O and analyzed via RT-qPCR on the same day. Each extraction included a negative control and a positive control used to estimate the virus recovery efficiency. Samples with a virus recovery ≥ 1% were considered acceptable.

### 2.4. Duplex Real-Time RT-qPCR Conditions

Viral RNA was detected via RT-qPCR on a Mx3000P qPCR system (Stratagene, La Jolla, CA, USA) instrument. SARS-CoV-2 RNA concentrations in wastewater samples were obtained using the N1 assay targeting a fragment of the nucleocapsid gene [32]. Primer/probe sets employed for the detection of the different viruses are listed in Table 1. Twist Synthetic SARS-CoV-2 RNAs (Twist Bioscience, San Francisco, CA, USA) were used as a positive control for wild type (Control 2—MN908947.3) and all VOC (Control 14—B.1.1.7_710528; Control 16—EPI_ISL_678597; Control 23—EPI_ISL_1544014; Control 48—EPI_ISL_6841980), and were also used to prepare standard curves for genome quantitation.

A RT-qPCR mastermix was prepared using PrimeScript™ One Step RT-PCR Kit (Takara Bio, London, UK). The reaction mix (10 μL) consisted of 3.35 μL of RNA-free H2O, 5 μL of 2 × One Step RTPCR Buffer III, 0.20 μL PrimeScript RT enzyme Mix II, 0.20 μL TaKaRa Ex Taq HS, 0.5 μL for each set of forward and reverse primers, and 0.25 μL for each probe. For the MHV-A59 and SARS-CoV-2 N1 region, the temperature program was set to 15 min at 50 °C, 3 min at 95 °C, and 45 cycles of 15 s at 95 °C and 30 s at 58 °C. The thermal cycling conditions for all duplex RT-qPCR assays were performed as described in Carcereny et al. [13,33] and MITECO [30]: RT at 50 °C for 10 min, preheating at 95 °C for 3 min and 45 cycles of amplification at 95 °C for 3 s and 60 °C for 30 s. The S gene of each VOC was analyzed using a duplex gene allelic discrimination TaqMan RT-qPCR assay, with 400 nM of the primers targeting the S gene and 200 nM of the probes (Table 1). Alpha, Beta, and Delta deletions are mapped to the S gene (residues 69/70, 242/244 and 157/158, respectively) (Table 1). The Omicron variant harbors a high number of mutations (more than 50), with 26–32 of them in the S-gene (including delHV69-70 and del143-145) (Table 1).

Each RNA was analyzed in duplicate and as pure (undiluted RNA) and ten-fold diluted to detect possible presence of inhibitors. Every RT-qPCR assay included four wells corresponding to negative controls (two nuclease-free water and two negative extraction controls), and one well with each corresponding synthetic SARS-CoV-2 RNA positive controls at 10^3^ copies/µL [13,30].

Synthetic RNA controls were quantified via droplet-based digital PCR using the One-Step RT-ddPCR Advanced Kit for Probes in a QX200TM System (Bio-Rad, Madrid, Spain) to estimate the exact concentration of GC/µL, prior to construction of RT-qPCR standard curves. Calibration curves for SARS-CoV-2 VOC RNAs were constructed using a minimum of five ten-fold dilutions and three wells for each dilution using two synthetic SARS-CoV-2 RNA controls corresponding to the variant containing the specific mutation and the variant without the specific mutation as described in Carcereny et al. [13,33] and MITECO [30].

### 2.5. Data Analysis and Interpretation

For each specific SARS-CoV-2 VOC target, Cq values ≤ 40 were converted into GC/L using the corresponding standard curve and volumes tested. The occurrence of inhibition was estimated by comparing average viral titers obtained from duplicate wells tested for the undiluted RNA with duplicate wells tested on ten-fold diluted RNA [13,30]. Inhibition was ascertained when difference in average viral titers was higher than 0.5 log_10_ and, in these cases, viral titers were inferred from the ten-fold RNA dilution. The percentage of SARS-CoV-2 genomes containing the respective mutation within the S gene were calculated as described in Carcereny et al. [13] and MITECO [33].

All statistical analysis and data plotting were performed with R Studio (version 4.3.1). The Kruskal–Wallis test was used to determine whether there were differences in viral loads of each VOC across the months, followed by Dunn’s test (post-hoc) for pairwise comparisons. Differences were considered statistically significant when *p*-values < 0.05.

The relative abundances of Alpha, Delta, and Omicron in wastewater samples were compared with prevalence data from clinical samples collected exclusively from individuals admitted to the Complexo Hospitalario Universitario de Santiago (CHUS; University Hospital Complex of Santiago). Based on the normality of the data, the Pearson correlation coefficient (*r*) or the Spearman’s rank correlation analysis (ρ) were employed to correlate the percentages of abundance of each VOC in wastewater with the percentages of abundance of each VOC in clinical samples.

### 2.6. Sequencing of Clinical Samples

Anonymized nasopharyngeal exudate samples putatively infected with SARS-CoV-2 were obtained at the Microbiology Service of the CHUS, which was the only hospital treating COVID-19 patients in the municipality. RNA from the nasopharyngeal exudates was extracted with an MagNA Pure LC extraction system kit (Roche, Sant Cugat, Spain), following the manufacturer’s recommendations. SARS-CoV-2 genome copy numbers in the samples were calculated via real-time RT-PCR of the N gene, and samples with Ct < 30 were subjected to sequencing (*n* = 1750).

Whole Genome Sequencing was performed using the ARTIC network’s PCR protocol, with the different primer pool versions adapted to “ARTIC n-CoV-2019”. Libraries were performed following Nextera DNA library preparation kit instructions (Illumina, San Diego, CA, USA). These libraries were barcoded with unique dual indexes, pooled, and sequenced using a NextSeq Reagent kit v.2 2 × 150 cycles (Illumina, San Diego, CA, USA) in a MiSeq sequencer (Illumina, San Diego, CA, USA).

## 3. Results

During this study’s 13-month period (January 2021–January 2022), a total of 127 samples from the WWTP inlet and 52 samples from each sewer interceptor (SI-1 and SI-2) were analyzed. Regarding recovery of the MHV used as process control virus, extraction efficiencies (mean ± standard deviation) were 13.8 ± 16.4% for WWTP samples, 12.8 ± 14.9% for SI-1 samples, and 7.8 ± 11.2% for SI-2 samples. The calibration curve for N1 was y = −3.473 ∗ Log x + 39.46, amplification efficiency (Eff.) = 94.1%, and correlation coefficient (R^2^) = 0.946. In the case of the N1 target, the limit of detection (LoD) and limit of quantification (LoQ) of the overall method for wastewater samples were 6.9 × 10^3^ and 2.4 × 10^4^ GC/L, respectively. The LoD, LoQ, and calibration curves for each S duplex RT-qPCR assay are described in Table 2.

### 3.1. Detection and Quantification of VOCs Surveillance in Samples from the WWTP Inlet

Among the 127 wastewater samples tested, 106 (83.5%) were positive for the N1 gene target, with concentrations ranging between ≤LOQ and 4.5 × 10^6^ GC/L (Supplementary Figure S2). Inhibition was observed in 30.2% of the samples. Alpha, Beta, Delta, and Omicron variants were detected in 45.7%, 7.5%, 66.7%, and 72.7% of samples, respectively. Inhibition was observed in 18.33% (11/60), 14.29% (1/7), 13.46% (7/52), and 37.50% (6/16) of WWTP samples for Alpha, Beta, Delta, and Omicron, respectively (Supplementary Table S1). A total of 93 wastewater samples were positive for at least one variant, 19 were found positive for at least two variants, while one sample was found positive for three variants. A significant moderate positive correlation was observed between the SARS-CoV-2 genome concentration measures for N1 and total S gene titers (ρ = 0.567).

Quantification levels along the study period ranged between 2.4 × 10^3^ and 2.5 × 10^6^ GC/L for the Alpha variant, between 2.2 × 10^3^ and 2.44 × 10^5^ GC/L for the Beta variant, between 2.2 × 10^3^ and 2.2 × 10^6^ GC/L for the Delta variant, and between 2.4 × 10^3^ and 4.0 × 10^6^ GC/L for the Omicron variant (Supplementary Table S1). Maximum quantification levels for Alpha (2.5 × 10^6^ GC/L) and Beta (2.4 × 10^5^ GC/L) were reached in January 2021, corresponding to the peak of third wave (Supplementary Table S1). The highest RNA levels for Delta (2.2 × 10^6^ GC/L) in WWTP samples were detected in July 2021, coincident with the peak of the fifth wave (Supplementary Table S1), and Omicron reached its highest quantification levels (4.0 × 10^6^ GC/L) in January 2022, when the sixth wave begun (Supplementary Table S1).

The mean viral load detected in WWTP samples via RT-qPCR for Omicron (6.1 × 10^5^ GC/L) was one-fold greater than that observed for Alpha (5.6 × 10^4^ GC/L) and Delta (9.4 × 10^4^ GC/L), and two-fold greater than that observed for Beta (4.8 × 10^3^ GC/L).

### 3.2. Detection and Quantification of VOCs Surveillance in Samples from Sector Interceptors

Analysis of samples from sewer interceptor 1 (SI-1) yielded a total of 88.5% (46/52) positive for the SARSCoV-2 N1 gene target, with concentrations ranging between 3.9 × 10^3^ and 3.3 × 10^6^ GC/L (Supplementary Figure S2). Partial amplification inhibition was observed in almost half of the samples (45.6%). Alpha was detected in 48.1% (25/52), whereas Beta, Delta, and Omicron variants were detected in 12.8% (5/39), 68.6% (24/35), and 71.4% (5/7). Inhibition of amplification observed in duplex RT-qPCR protocols ranged from 20% (Alpha, Beta, and Omicron) to 29% (Delta) of the samples (Supplementary Table S2). Thirty-four samples were positive for at least one variant, 11 were found positive for at least two variants, and one sample was found positive for three variants. A significant strong positive correlation was observed between the SARS-CoV-2 genome concentration measures for N1 and total S gene titers (ρ = 0.694).

Quantification levels ranged between 2.1 × 10^3^ and 2.3 × 10^6^ GC/L for Alpha, 2.0 × 10^3^ and 1.9 × 10^6^ GC/L for Beta, 5.4 × 10^3^ and 1.8 × 10^6^ GC/L for Delta, and 4.4 × 10^3^ and 9.3 × 10^5^ GC/L for Omicron (Supplementary Table S2). The highest quantification levels for each variant were reached in January 2021 for Alpha and July 2021 for Beta and Delta. Omicron RNA levels increases throughout December 2021 (Supplementary Table S2), but unfortunately no samples from sewer interceptors were obtained in January 2022. Although quantification ranges for all VOCs were similar, mean viral loads for Alpha (1.4 × 10^5^ GC/L), Delta (1.6 × 10^5^ GC/L), and Omicron (2.4 × 10^5^ GC/L) VOCs were approximately 0.5-fold greater than that observed for the Beta variant (6.2 × 10^4^ GC/L).

For samples from sewer interceptor 2 (SI-2), 94.2% (49/52) were positive for the N1 gene target, and concentrations ranged from 3.8 × 10^3^ and 3.8 × 10^6^ GC/L (Supplementary Figure S3), with inhibition being observed in 46.9% (23/49) of samples. For SI-2 samples, 48.1%, 7.7%, 80.0%, and 42.9% were positive for Alpha, Beta, Delta, and Omicron variants, respectively. Inhibition in the duplex RT-qPCR was higher than that observed for samples from SI-1, ranging from 24% (Alpha) to 66.7% (Omicron) (Supplementary Table S3). Thirty-six samples were positive for one variant and 11 were found positive for two variants. A significant strong positive correlation was observed between the SARS-CoV-2 genome concentration measures for N1 and total S gene titers (ρ = 0.609).

Quantification levels ranged between 2.2 × 10^3^ and 5.2 × 10^6^ GC/L for Alpha, 1.6 × 10^3^ and 7.1 × 10^5^ GC/L for Beta, 2.5 × 10^3^ and 4.8 × 10^6^ GC/L for Delta, and 2.4 × 10^5^ and 4.8 × 10^6^ GC/L for Omicron (Supplementary Table S3). Mean viral loads for Beta (1.9 × 10^4^ GC/L) were at least one-fold lesser than total viral loads for Alpha (1.8 × 10^5^ GC/L), Delta (3.2 × 10^5^ GC/L), and Omicron (8.0 × 10^5^ GC/L) variants. Alpha reached its highest quantification levels in February 2021, Beta in April, and Delta in November 2021 (Supplementary Table S3). As for SI-1, Omicron RNA levels increased throughout December 2021 (Supplementary Table S3), and no samples were analyzed in January 2022.

### 3.3. Temporal Evolution of VOC in Wastewater

**Figure 2:**
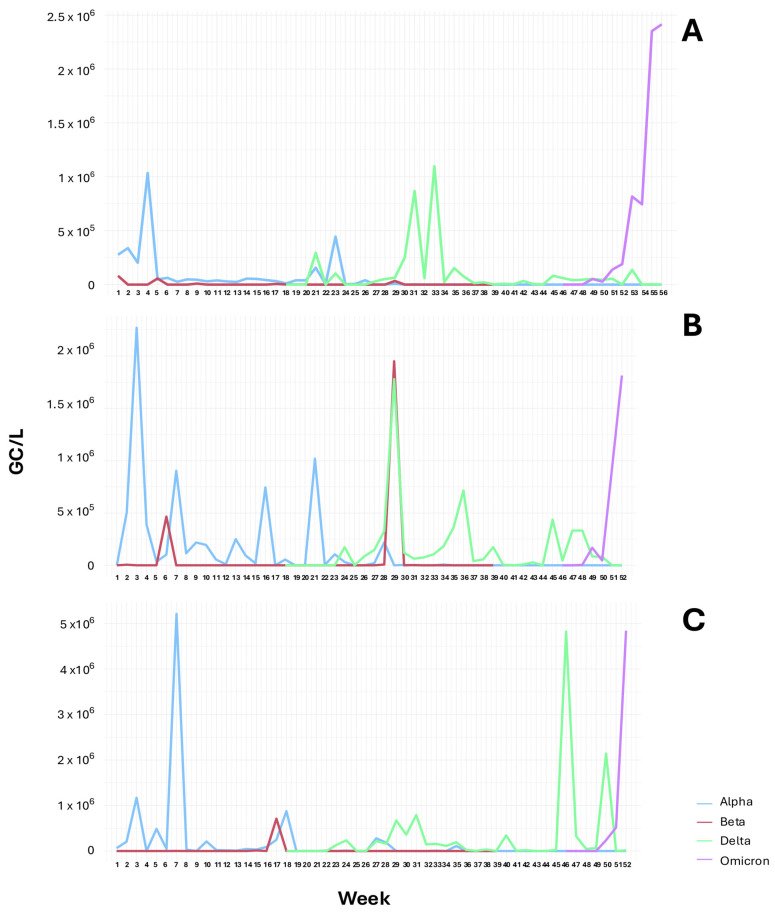
Mean quantification levels (GC/L) of Alpha, Beta, Delta, and Omicron VOCs per week (56 weeks) along the study in the WWTP (**A**), SI-1 (**B**), and SI-2 (**C**) samples analyzed.

**Figure 3:**
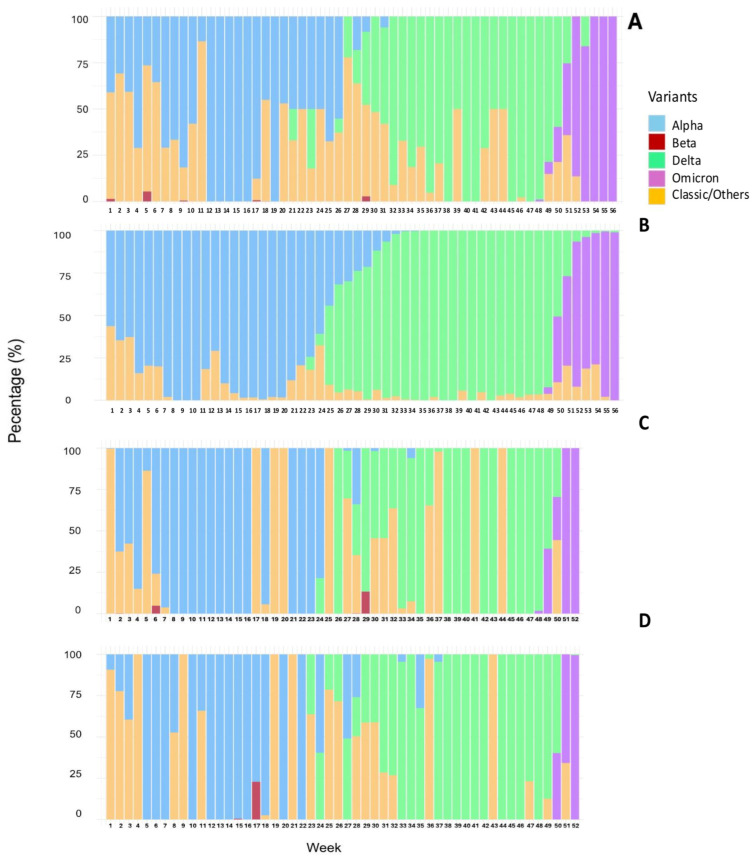
Comparison of VOC proportion (%) along the study in WWTP samples (**A**), SI-1 (**B**), SI-2 (**C**), and clinical cases (**D**) in the area of Santiago de Compostela on a weekly basis.

The temporal evolution of each VOC in the WWTP, as well as in sewer interceptors (Figure 2 and Figure 3; Supplementary Tables S1–S6), reveals the clear dominance of Alpha in the first 6 months of the study (January–June 2021), followed by Delta (July–November 2021), and Omicron (December 2021–January 2022). The Beta variant was detected only sporadically during the study period.

Figure 2A shows the viral concentrations over 56 consecutive weeks in wastewater samples collected at the WWTP. H69/V70 deletion in the Alpha variant appeared at the beginning of our study in early January 2021 (from week 1), and during this month, this mutation reached its highest RNA levels in the WWTP. Then, from week 6, a decrease in B.1.1.7 RNA levels was observed, although this variant continued to be detected over the following months until the beginning of August 2021 (week 29) (Figure 2A). Beta VOC was only detected in weeks 1, 5, 9, 17–18, and 29 (Figure 2A), and these detections were coincident with high concentrations of the SARS-CoV-2 N1 gene fragment (see Supplementary Figure S2). The Delta variant was first observed in week 21 and consistently detected from week 26 to week 51 (December 2021), reaching maximum levels between week 29 (July 2021) and week 36 (September 2021) (Figure 2A). Omicron was first detected at the end of November (week 49), and its concentration continuously increased until the end of our study in late January 2022 (week 55), when the highest RNA concentration of this VOC was reached (Figure 2A).

Significant differences (*p*-value < 0.05) were observed in the concentration of the Alpha variant over the 13 months during which this VOC was analyzed. Alpha concentration levels decreased significantly (*p*-value < 0.05) between July 2021 and January 2022 compared to the levels observed at the beginning of the study (January 2021). Delta quantification levels showed a significant increase (*p*-value < 0.05) in July and August 2021 compared to May and June 2021. This notable rise in Delta concentration occurred concurrently with the significant decrease in Alpha levels mentioned earlier. From September 2021 to January 2022, no significant changes were observed in Delta levels. Omicron concentration levels were significantly higher (*p*-value < 0.05) in January 2022 in comparison with November and December 2021. No significant differences were observed in the concentration of the Beta variant in WWTP samples or in SI-1 and SI-2 samples throughout the 9 months during which this VOC was analyzed.

As mentioned above, the general trends of the different VOCs were the same for the sewer interceptors, although some divergences were observed (Figure 2 and Figure 3). Thus, the highest Alpha concentrations were detected in week 3 for SI-1, with notable increments in weeks 7, 16, and 20 (Figure 2B). For SI-2, the highest Alpha levels were observed in February 2021 (week 7) (Figure 2C). Two peaks of Beta VOC were observed at SI-1 in weeks 6 and 29, the latter being coincident with the highest levels of the Delta Variant (Figure 2B), whereas the Beta variant was detected in SI-2 in week 17 (Figure 2C). Two important increases for Delta were observed at SI-2 during weeks 46 and 50, which were not observed with that magnitude at SI-1 (Figure 2B,C). Finally, although the first detections of Omicron were observed at the same time in both interceptors, increases in its concentration occurred faster in SI-1 (Figure 2B,C).

In SI-1 and SI-2, Alpha concentration levels were significantly reduced (*p*-value < 0.05) from September to December 2021 compared to the levels observed at the beginning of the study (January and February 2021). Delta quantification levels increased significantly from July 2021 onwards, compared to May 2021. However, no significant differences were observed for this variant in the second half of the year. Again, Omicron concentration levels were significantly higher (*p*-value < 0.05) in January 2022 in comparison with November and December 2021, which showed no significant differences between them.

### 3.4. VOC Proportion and Comparison with Clinical Cases

In parallel with the comparison of VOC concentrations across months, we also examined the variations in their proportions over the same period in WWTP samples (Figure 3; Supplementary Tables S4–S6). Alpha abundance showed a significant decrease (*p*-value < 0.05) from July 2021 to January 2022. The proportion of the Delta variant increased significantly (*p*-value < 0.05) in August–November compared to May and June 2021. Furthermore, the Delta proportion significantly decreased (*p*-value < 0.05) in January 2022 compared to the second half of 2021. The proportion of Omicron increased significantly (*p*-value < 0.05) in January 2022 compared to November and December 2021. Similarly to concentration levels, no significant differences were observed in the proportion of the Beta variant in WWTP samples across the 9 months during which this VOC was analyzed.

The proportions of Alpha, Beta, Delta, and Omicron variants in the samples analyzed from SI-1 and SI-2 followed trends consistent with those observed in wastewater (Figure 3B,C).

The proportion of each VOC in WWTP samples was compared to the prevalence detected at the clinical level, observing similar trends and a good correlation for the emergence and local spread of these VOCs (Figure 3). Significant strong positive correlations were observed between the percentages of abundance of the Alpha and Delta variants in wastewater and in clinical samples (ρ = 0.868 and 0.785, respectively). Also, a significant strong positive correlation was observed between the percentages of abundance of the Omicron variant in wastewater and in clinical samples (*r* = 0.963). It is interesting to note that the diversity of VOCs detected in wastewater was higher than that observed in clinical samples. In fact, the Beta variant was detected with lower prevalence (never higher than 3%) at different periods in wastewater samples but was never detected in clinical samples (Figure 3).

At the beginning of the study (weeks 1–8), the Alpha variant represented between 35 and 70% within the WWTP positive samples, being the only variant detected between weeks 12 and 16. From this week, proportion of Alpha was slowly declining until week 31, not being detected beyond that date (Figure 3A). For clinical samples, percentages of the Alpha variant ranged between 56.3 and 100% from week 3 to week 25, decreasing later until week 35, the last day with positive detection of this VOC (Figure 3D; Supplementary Table S7).

The Delta variant was detected in wastewater three weeks earlier than in clinical cases (Figure 3). The B.1.617.2 variant was detected for the first time in week 21 (16.9% of relative abundance) in the WWTP. From this point, Delta was progressively and rapidly displacing the Alpha variant until it became the dominant variant (100%) in the WWTP in week 38 (Figure 3A). Delta appearance in clinical samples occurred in week 24 with 7.7%. Then, an important increase was observed in week 27, reaching values around 60%. In week 34 and until week 49, the Delta variant was clearly dominant in clinical cases corresponding with more than 90% of the samples sequenced (Figure 3D; Supplementary Table S7).

The Omicron variant was also detected earlier in wastewater than in clinical samples. The first detection in the WWTP occurred in week 48, with a proportion of 5% (Figure 3A). Omicron displaced Delta very fast, and by week 52 the proportion of this variant in wastewater was higher than 80% (100% from week 54). Omicron appeared in clinical samples in week 49, with a relative abundance of 3.8%, while Delta was the dominant variant, accounting for more than 92% of clinical sequences and reached values higher than 80% only in week 55 (Figure 3D; Supplementary Table S7).

## 4. Discussion

From the beginning of the COVID-19 pandemic, genomic surveillance based on sequencing individual clinical samples (nasal swabs) was very effective for tracking the emergence of new variants, and also to model future waves [34]. Soon after the first cases, the presence of virions was demonstrated in the feces of patients infected with SARS-CoV-2, as well as in wastewaters, leading to the development of procedures to be applied under an environmental surveillance approach. The integration of microbiological and environmental surveillance systems constituted an adequate strategy for the early detection of viral spread and epidemiological studies [35]. In fact, the WHO has released a guide for public-health decision-makers to integrate environmental surveillance of wastewater into COVID-19 monitoring control strategies [34].

In the present study, we monitored the emergence and spread of SARS-CoV-2 VOCs (mainly B.1.1.7, B.1.617.2, and B.1.1.529) in Santiago de Compostela for a period of 13 months during 2021 and the beginning of 2022. According to official data from the Ministry of Health (https://www.sanidad.gob.es/, accessed on 17 March 2025), this period encompassed the rise of four COVID-19 waves (Spanish waves 3 to 6). The results obtained were compared with those from clinical samples from the University Hospital Complex of Santiago (CHUS).

Three clear increases in SARS-CoV-2 RNA levels were detected in wastewater from the WWTP in January–February 2021, August 2021, and December 2021–January 2022, which corresponded with the dominance of Alpha, Delta, and Omicron VOCs, respectively. Such increases were strongly associated with the rise of the third, fifth, and sixth wave in Spain (https://cnecovid.isciii.es/covid19/#evoluci%C3%B3n-de-la-pandemia; accessed on 26 March 2024). A fourth minor peak was observed around weeks 20–24 in which co-dominance of Alpha and Delta was detected, which was related with the emergence of the fourth wave (which was known as the little wave in Spain). Trends in RNA levels were very similar for SI-2, covering a neighborhood with stable local population. However, RNA levels in the sewer interceptor SI-1, associated with the historic center of the town, showed higher variability. Although the dominance of the different variants was in concordance with the other sampling points, periodic minor increases were observed throughout the year. The Beta variant was only detected in a few samples from the WWTP, SI-1, and SI-2 at different times between January and August 2021 and in a much lower proportion than the other VOCs. Our results are in agreement with those obtained by Agrawal et al. [16], who also detected B.1.351 in wastewater samples in Spain at a very low frequency. It is noteworthy that the total viral loads detected for the Omicron variant were greater than those observed for the Alpha, Beta, and Delta variants, an observation made previously in other geographic areas [24,28].

The affluence of national and international visitors, mainly to the Historic City Center, may explain in part the higher variability observed in the results from SI-1 as well as the several peaks of the Beta variant detected in samples from this neighborhood. A previous study by Gallego-García and coworkers [36], analyzing the SARS-CoV-2 introductions in our region, inferred independent introductions of the Alpha variant from different Spanish regions and also from France and Portugal. For the Delta variant, the most significant sources were Portugal, USA, France, and Germany, whereas for Omicron they were France, Portugal, USA, Germany, and UK. Such findings support well our hypothesis that international visitors could influence the presence of the different variants in the Historic City Center at Santiago de Compostela.

As in previous studies [13,14,15,16,18,19,20,21,22,25,26], RNA concentrations of Alpha, Delta, and Omicron VOCs in wastewater has been found to be positively associated with the clinical appearance of these VOCs in the area of Santiago. Regarding the Beta variant, clinical cases were not reported by CHUS, so its detection in wastewater constitutes the first notification of this variant in Santiago de Compostela.

Thus, the Alpha variant was the predominant variant in Santiago de Compostela wastewater since the beginning of our study (January 2021) until the appearance of the Delta variant. The first appearance of Delta occurred in the WWTP in week 21 (24–27 May 2021), 3 weeks prior to detection through clinical genomic surveillance. Displacement of the Alpha variant took longer in wastewater (approx. 5–6 weeks) than in clinical samples (3–4 weeks), indicating its greater transmissibility and infectivity. Delta predominated until Omicron emergence in week 48 (29 November–2 December), only one week before its clinical detection. Displacement of Delta by Omicron was much faster than that observed between Alpha and Delta. Thus, in only 1–3 weeks Omicron was the dominant variant both in wastewater and clinical samples. The observed evolution of VOCs in Santiago de Compostela agrees with that observed in previous studies in different countries, where displacement by Delta and Omicron followed a similar trend and rapidity [14,15,17,18,19,20,21,22,24,26,28].

The utility of viral load in wastewater as a leading indicator for clinical data has been extensively described, with the lag phase between virus detection in wastewater and the appearance of clinical cases ranging from 1 day to 2 weeks [37,38]. Such correlation and capacity of prediction are influenced by environmental factors, epidemiological conditions, sampling design and catchment population [39]. Another key factor is analytical effort, since an increase in the sampling frequency together with a high level of sequencing can improve the correlation, namely for VOCs. In our study, the sequencing effort for clinical samples increased from 30 samples per week at the beginning of 2021 up to 50 per week at the end of the year. This fact, together with the huge viral loads detected for the Omicron variant could explain the shorter lag time for this variant.

We are conscious of some limitations in the present study, including the use of different analytical approaches for wastewater (VOC-targeted duplex RT-PCR) and clinical samples (sequencing), as well as the selection of specific neighborhoods instead of a broader sampling design in the city. With respect to the analytical choice, sequencing has also been applied to wastewater, allowing the detection of multiple mutations and variants, although processing of the wastewater samples for this approach is more complicated and the technique itself more expensive [1,3,5,20]. The good correlation observed in our studies indicated that no bias was introduced by using different approaches for the two sample types. Regarding the selection of city neighborhoods, it would be interesting to analyze more city sectors, including rural sectors, to get a deeper epidemiological picture. Selection was made according to population size and the influence of tourism as factors affecting viral transmission and the emergence of new variants.

In summary, the results obtained in the present work highlight the applicability of real-time RT-PCR to track the emergence and spread of SARSCoV-2 VOCs (mainly B.1.1.7, B.1.617.2, and B.1.1.529) in Santiago de Compostela through routine screening of the wastewater surveillance network, providing early alerts 1 to 4 weeks before the emergence and rise of new VOCs in clinical samples. Therefore, this approach proved to be a useful complement to clinical testing, providing additional information about the evolution of the pandemic that can be crucial for implementation of preventive measures by public health authorities.

## Figures and Tables

**Table 1:** List of primers and probes used for all RT-qPCR assays [13,33].

SARS-CoV-2 VOC	Name	Sequence (5′–3′) ^1^	Tm (°C)
Alpha	For-S21708	ATTCAACTCAGGACTTGTTCTTACCTT	64.1 °C
	Rev-S21796	TAAATGGTAGGACAGGGTTATCAAAC	63.7 °C
	S_Probe6970in	FAM/TCCATGCTATACATGTCTCTGGGACCAATG/BHQ1	73.8 °C
	S_Probe6970del	HEX/TTCCATGCTATCTCTGGGACCAATGGTACT/BHQ1	73.3 °C
Beta	For-S22239	TGGTAGATTTGCCAATAGGTATTAACA	54.3 °C
	Rev-S22307	TGAAGAAGAATCACCAGGAGTCAA	55.8 °C
	S_Probe242_244in	FAM/ACTTTA+CTT+G+CT+TTACA+TA+GAAG/IABkFQ	59.8 °C
	S_Probe242_244del	HEX/CTAG+GTTT+CAAACTTTACAT+AGAAG/BHQ1	54.0 °C
Delta	For-S157158v2	TTACCACAAAAACAACAAAAGTTGG	53.3 °C
	Rev-S157158v2	GCTGAGAGACATATTCAAAAGTGCAA	55.8 °C
	S_Probe157158v2	FAM/TGGAAA+G+T+GGAGTTTATT+C+TAGTG/IABkFQ	61.4 °C
	S_Probe157158del	HEX/AGT+GAGTTCA+G+AGTTTATT+CTA+GTG/IABkFQ	61.0 °C
Omicron	For-Omicron	AGGAAAACAGGGTAATTTCAAAAATC	52.7 °C
	Rev-Omicron	CCAATGGTTCTAAAGCCGAAAA	53.8 °C
	S_ProbeOmicron	FAM/TGCGTGAGCCAGAAGATCTCCCTCA/IABkFQ	63.4 °C
	S_ProbeNoOmicron	HEX/CGCCTATTAATTTAGTGCGTGATCTCCCTCA/IABkFQ	61.1 °C

^1^ +A, +G, +C, +T indicate locked nucleic acids (LNA).

**Table 2:** Calibration curves and limits of detection (LoD) and quantification (LoQ) (genome copies/liter, GC/L) for each S duplex RT-qPCR assay.

Target	Slope	Intercept	Efficiency (%)	R^2^	LoD (GC/L)	LoQ (GC/L)
S_Probe6970in	−3.729	41.45	85.4	0.965	1.0 × 10^3^	2.9 × 10^3^
S_Probe6970del	−3.429	41.29	95.7	0.989	7.5 × 10^2^	1.1 × 10^3^
S_Probe242_244in	−3.725	42.58	85.5	0.821	1.2 × 10^3^	3.9 × 10^3^
S_Probe242_244del	−3.408	40.14	96.5	0.944	2.2 × 10^2^	4.8 × 10^2^
S_Probe157158v2	−3.705	40.87	86.2	0.915	9.3 × 10^2^	2.3 × 10^3^
S_Probe157158del	−3.680	40.91	87.0	0.992	9.8 × 10^2^	2.0 × 10^3^
S_ProbeOmicron	−3.636	37.16	88.4	0.998	9.8 × 10^2^	1.3 × 10^3^
S_ProbeNoOmicron	−3.522	38.86	92.3	0.976	1.6 × 10^3^	2.3 × 10^3^

## Data Availability

Genome sequences and associated metadata are available in GISAID’s EpiCoV database (https://gisaid.org).

## Supplementary Materials

The following supporting information can be downloaded at: https://www.mdpi.com/article/10.3390/v17040489/s1, Figure S1: Official number of pilgrims with national and international origin arriving in Santiago de Compostela during the period of study Figure S2: Quantification levels (GC/L) of N1 along the study in all WWTP, SI-1, and SI-2 samples analyzed; Tables S1–S3: Quantification levels (GC/L) of Alpha, Beta Delta and Omicron VOCs along the study in WWTP, SI-1, and SI-2 samples; Tables S4–S6: Temporal changes in the detection frequency (%) of VOCs in wastewater from WWTP, SI-1, and SI-2 using RT-qPCR.; Table S7: VOC proportion (%) in the clinical samples analyzed.
