# Supplementary material for: Monitoring the Emergence of SARS-CoV-2 VOCs in Wastewater and Clinical Samples—A One-Year Study in Santiago de Compostela (Spain)
Source: Viruses. 2025 Mar 28;17(4):489. doi: 10.3390/v17040489 (PMC12030845; doi:10.3390/v17040489)
Supplement: Supplementary file 1 [file viruses-17-00489-s001.zip › viruses-3481805-supplementary.pdf]

SUPPLEMENTARY MATERIAL

Monitoring the emergence of SARS-CoV-2 VOCs in wastewater and clinical samples. A one-year study in Santiago de Compostela (Spain)

Marta Lois[1], David Polo[1], María Luisa Pérez del Molino[2], Amparo Coira[2], Antonio Aguilera[2] and Jesús L. Romalde[1]

[1] Department of Microbiology and Parasitology, CIBUS-Facultade de Bioloxía & CRETUS, Universidade de Santiago de Compostela, 15782 Santiago de Compostela, Spain.

[2] Servicio de Microbiología, Hospital Clínico Universitario de Santiago, Santiago de Compostela, Spain.

Supplementary Figure S1. Official number of pilgrims with national and international origin arriving in Santiago de Compostela during the period of study. Data from https://oficinadelperegrino.com/en/statistics-2/.

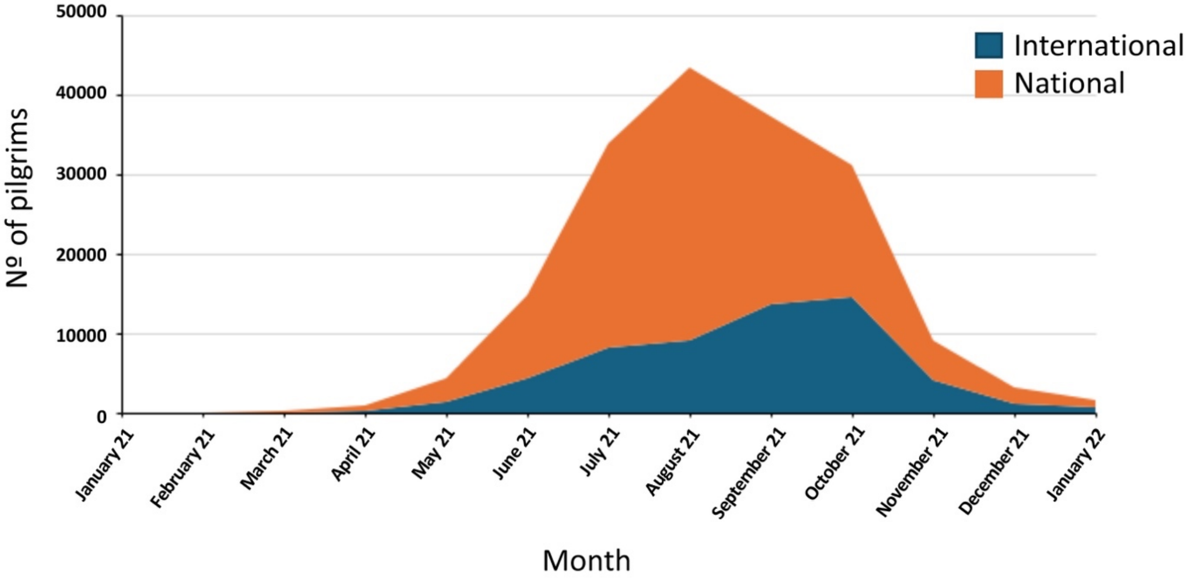

Supplementary Figure S2. Quantification levels (GC/L) of N1 in WWTP (A), SI-1 (B) and SI-2 (C) samples analyzed. along the study.

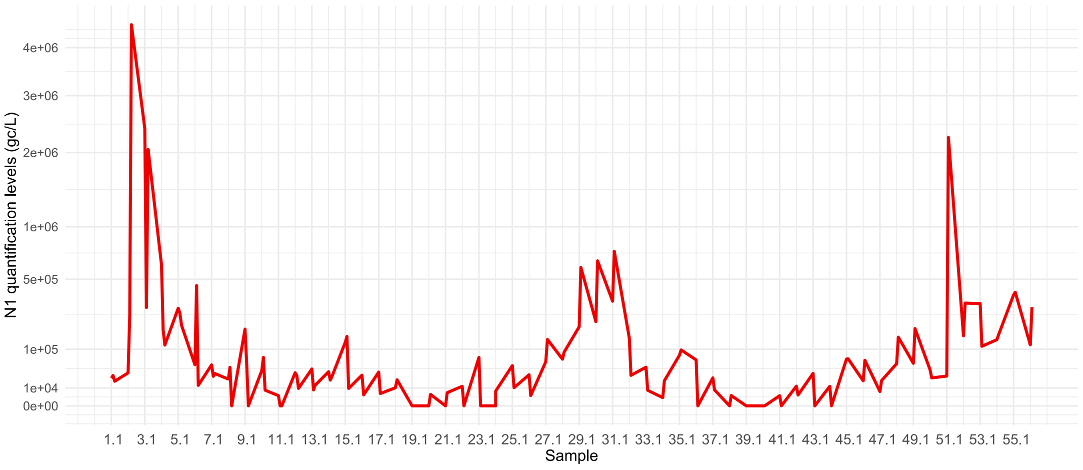

A

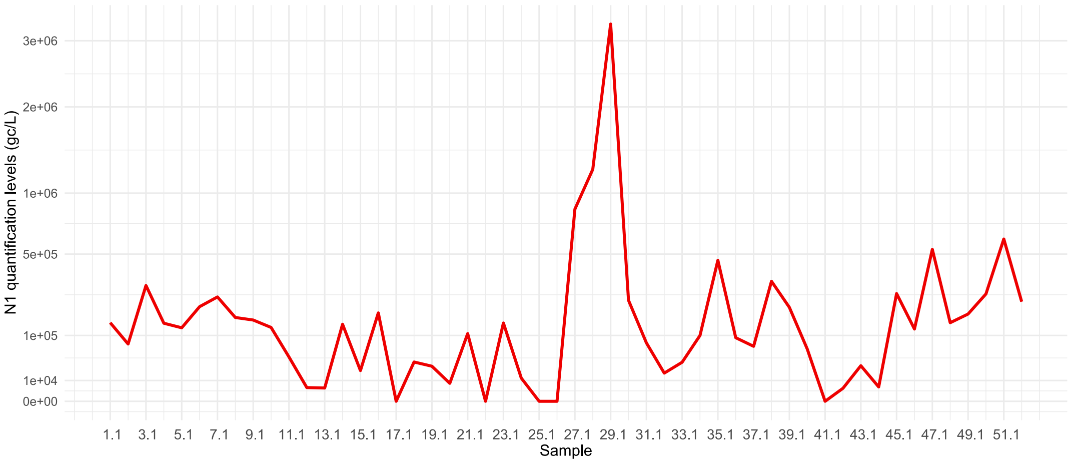

B

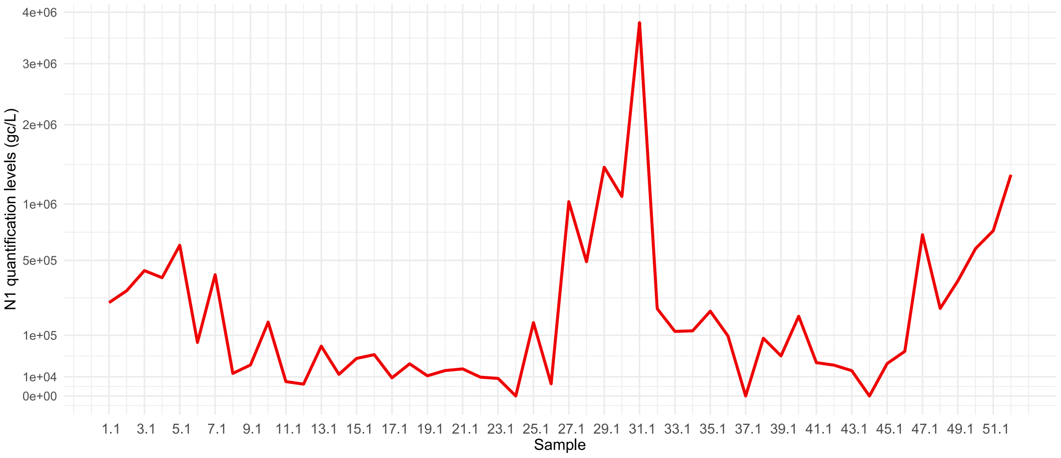

C

Supplementary Table S1. Quantification levels (gc/L) of Alpha, Beta, Delta and Omicron VOCs along the study in all WWTP samples analyzed.

Week	Sample	Alpha	Beta	Delta	Omicron
1	1.1	5,46E+05	0,00E+00		
	1.2	1,21E+05	2,44E+05		
	1.3	1,64E+05	0,00E+00		
2	2.1	3,92E+04	0,00E+00		
	2.2	0,00E+00	0,00E+00		
	2.3	9,76E+05	0,00E+00		
3	3.1	5,83E+04	0,00E+00		
	3.2	1,13E+05	0,00E+00		
	3.3	4,36E+05	0,00E+00		
4	4.1	2,52E+06*	0,00E+00		
	4.2	4,27E+05	0,00E+00		
	4.3	1,58E+05	0,00E+00		
5	5.1	1,06E+05*	0,00E+00		
	5.2	3,79E+04*	1,69E+05		
	5.3	0,00E+00	0,00E+00		
6	6.1	3,15E+04	0,00E+00		
	6.2	1,56E+05	0,00E+00		
	6.3	0,00E+00	0,00E+00		
7	7.1	2,81E+04*	0,00E+00		
	7.2	3,02E+04	0,00E+00		
	7.3	2,02E+04	0,00E+00		
8	8.1	1,71E+04	0,00E+00		
	8.2	0,00E+00	0,00E+00		
	8.3	1,29E+05	0,00E+00		
9	9.1	9,57E+04*	2,71E+04*		
	9.2	3,23E+04	0,00E+00		
	9.3	6,48E+03	0,00E+00		
10	10.1	2,64E+03	0,00E+00		
	10.2	8,75E+04	0,00E+00		
	10.3	0,00E+00	0,00E+00		
	11.1	7,75E+03	0,00E+00		

11	11.2	1,08E+05	0,00E+00		
	11.3	0,00E+00	0,00E+00		
12	12.1	3,51E+04*	0,00E+00		
	12.2	4,09E+04	0,00E+00		
	12.3	1,18E+04	0,00E+00		
13	13.1	1,37E+04	0,00E+00		
	13.2	4,72E+04*	0,00E+00		
	13.3	1,16E+04	0,00E+00		
14	14.1	5,60E+04	0,00E+00		
	14.2	1,14E+04	0,00E+00		
	14.3	9,80E+04	0,00E+00		
15	15.1	9,20E+04	0,00E+00		
	15.2	5,71E+04	0,00E+00		
	15.3	9,55E+03	0,00E+00		
16	16.1	3,78E+04	0,00E+00		
	16.2	4,61E+04*	0,00E+00		
17	17.1	4,16E+04	1,13E+04		
	17.2	2,17E+04	0,00E+00		
18	18.1	0,00E+00	0,00E+00	0,00E+00	
	18.2	2,25E+04	2,24E+03	0,00E+00	
19	19.1	7,56E+04*	0,00E+00	0,00E+00	
	19.2	3,93E+03	0,00E+00	0,00E+00	
20	20.1	0,00E+00	0,00E+00	0,00E+00	
	20.2	7,87E+04	0,00E+00	0,00E+00	
21	21.1	3,13E+05	0,00E+00	0,00E+00	
	21.2	0,00E+00	0,00E+00	5,89E+05	
22	22.1	7,24E+03	0,00E+00	0,00E+00	
	22.2	0,00E+00	0,00E+00	0,00E+00	
23	23.1	8,90E+05*	0,00E+00	0,00E+00	
	23.2	0,00E+00	0,00E+00	2,07E+05	
24	24.1	0,00E+00	0,00E+00	0,00E+00	
	24.1	7,02E+03	0,00E+00	0,00E+00	
25	25.1	4,33E+03	0,00E+00	0,00E+00	
	25.2	8,26E+03	0,00E+00	0,00E+00	

26	26.1	7,60E+04*	0,00E+00	0,00E+00	
	26.2	4,67E+03	0,00E+00	2,34E+03	
27	27.1	0,00E+00	0,00E+00	2,17E+04	
	27.2	0,00E+00	0,00E+00	3,72E+04	
28	28.1	0,00E+00	0,00E+00	2,84E+04	
	28.2	2,39E+03	0,00E+00	7,51E+04	
29	29.1	0,00E+00	2,70E+03	8,64E+04	
	29.2	1,48E+04	6,48E+04	4,04E+04	
30	30.1	0,00E+00	0,00E+00	2,77E+05*	
	30.2	0,00E+00	0,00E+00	2,24E+05	
31	31.1	0,00E+00	0,00E+00	1,65E+05	
	31.2	3,59E+03	0,00E+00	1,57E+06*	
32	32.1	0,00E+00	0,00E+00	7,34E+04	
	32.2	0,00E+00	0,00E+00	4,57E+04*	
33	33.1	0,00E+00	0,00E+00	2,19E+06*	
	33.2	0,00E+00	0,00E+00	6,42E+03	
34	34.1	0,00E+00	0,00E+00	1,00E+04	
	34.2	0,00E+00	0,00E+00	3,76E+04	
35	35.1	0,00E+00	0,00E+00	1,45E+05	
	35.2	0,00E+00	0,00E+00	1,61E+05	
36	36.1	0,00E+00	0,00E+00	7,64E+04	
	36.2	0,00E+00	0,00E+00	6,83E+04	
37	37.1	0,00E+00	0,00E+00	8,59E+03	
	37.2	0,00E+00	0,00E+00	1,93E+04	
38	38.1	0,00E+00	0,00E+00	3,19E+04	
	38.2	0,00E+00	0,00E+00	1,10E+04	
39	39.1	0,00E+00	0,00E+00	0,00E+00	
	39.2	0,00E+00	0,00E+00	2,84E+03	
40	40.1	0,00E+00		2,30E+03	
	40.2	0,00E+00		1,37E+04	
41	41.1	0,00E+00		2,24E+03	
	41.2	1,14E+05		3,53E+03	
42	42.1	0,00E+00		6,57E+04	
	42.2	1,37E+06		0,00E+00	

43	43.1	0,00E+00		1,04E+04	
	43.2	0,00E+00		0,00E+00	
44	44.1	0,00E+00		2,56E+03	
	44.2	0,00E+00		0,00E+00	
45	45.1	0,00E+00		1,56E+05*	
	45.2	0,00E+00		8,23E+03	
46	46.1	0,00E+00		2,46E+03	0,00E+00
	46.2	0,00E+00		1,18E+05	0,00E+00
47	47.1	0,00E+00		6,64E+03	0,00E+00
	47.2	0,00E+00		7,44E+04	0,00E+00
48	48.1	0,00E+00		4,31E+04	2,45E+03
	48.2	0,00E+00		4,79E+04	0,00E+00
49	49.1	0,00E+00		8,18E+04	6,52E+04
	49.2	0,00E+00		2,39E+04	3,82E+04*
50	50.1	0,00E+00		5,68E+04	5,11E+04
	50.2	0,00E+00		3,06E+04	3,92E+03
51	51.1	0,00E+00		7,55E+04	1,63E+04
	51.2	0,00E+00		3,42E+04*	2,62E+05
52	52.1	0,00E+00		0,00E+00	5,85E+04
	52.2	0,00E+00		0,00E+00	3,22E+05
53	53.1	0,00E+00		6,25E+04	7,25E+05*
	53.2	0,00E+00		2,11E+05*	9,09E+05
54	54.1	0,00E+00		0,00E+00	1,24E+06
	54.2	0,00E+00		0,00E+00	2,50E+05*
55	55.1	0,00E+00		6,01E+03	4,01E+06*
	55.2	0,00E+00		0,00E+00	6,94E+05
56	56.1	0,00E+00		0,00E+00	2,34E+06*
	56.2	0,00E+00		0,00E+00	3,49E+06*

* inhibition

Supplementary Table S2. Quantification levels (gc/L) of Alpha, Beta, Delta and Omicron VOCs along the study in all SI-1 samples analyzed.

Week	Sample	Alpha	Beta	Delta	Omicron
1	1.1	2,06E+03	0,00E+00		
2	2.1	5,07E+05	5,56E+03		
3	3.1	2,27E+06	0,00E+00		
4	4.1	3,88E+05	0,00E+00		
5	5.1	3,50E+04	0,00E+00		
6	6.1	1,02E+05	4,66E+05*		
7	7.1	9,04E+05	0,00E+00		
8	8.1	1,15E+05	0,00E+00		
9	9.1	2,18E+05*	0,00E+00		
10	10.1	1,95E+05	0,00E+00		
11	11.1	5,58E+04	0,00E+00		
12	12.1	1,03E+04	0,00E+00		
13	13.1	2,50E+05	0,00E+00		
14	14.1	9,39E+04*	0,00E+00		
15	15.1	1,81E+04	0,00E+00		
16	16.1	7,45E+05*	0,00E+00		
17	17.1	0,00E+00	0,00E+00		
18	18.1	5,49E+04	0,00E+00	0,00E+00	
19	19.1	0,00E+00	0,00E+00	0,00E+00	
20	20.1	0,00E+00	0,00E+00	0,00E+00	
21	21.1	1,02E+06*	0,00E+00	0,00E+00	
22	22.1	3,77E+03	0,00E+00	0,00E+00	
23	23.1	1,05E+05	0,00E+00	0,00E+00	
24	24.1	3,12E+04	0,00E+00	1,73E+05	
25	25.1	0,00E+00	0,00E+00	0,00E+00	
26	26.1	0,00E+00	0,00E+00	8,68E+04	
27	27.1	2,05E+04	0,00E+00	1,47E+05	
28	28.1	2,20E+05*	7,16E+03	3,27E+05	
29	29.1	0,00E+00	1,95E+06	1,78E+06	
30	30.1	3,39E+03	0,00E+00	1,20E+05	
31	31.1	0,00E+00	2,05E+03	6,29E+04	

32	32.1	0,00E+00	0,00E+00	7,55E+04	
33	33.1	0,00E+00	0,00E+00	1,06E+05*	
34	34.1	7,15E+03	0,00E+00	1,83E+05	
35	35.1	0,00E+00	0,00E+00	3,62E+05	
36	36.1	0,00E+00	0,00E+00	7,15E+05*	
37	37.1	0,00E+00	0,00E+00	4,05E+04	
38	38.1	0,00E+00	0,00E+00	5,64E+04	
39	39.1	0,00E+00	0,00E+00	1,74E+05*	
40	40.1	0,00E+00		5,44E+03	
41	41.1	0,00E+00		0,00E+00	
42	42.1	0,00E+00		7,07E+03	
43	43.1	0,00E+00		2,86E+04*	
44	44.1	0,00E+00		0,00E+00	
45	45.1	0,00E+00		4,37E+05*	
46	46.1	0,00E+00		4,57E+04	0,00E+00
47	47.1	0,00E+00		3,32E+05*	0,00E+00
48	48.1	0,00E+00		3,32E+05*	4,43E+03
49	49.1	0,00E+00		8,41E+04	1,68E+05
50	50.1	0,00E+00		8,15E+04	4,60E+04
51	51.1	0,00E+00		0,00E+00	9,28E+05*
52	52.1	0,00E+00		0,00E+00	1,81E+06

* inhibition

Supplementary Table S3. Quantification levels (gc/L) of Alpha, Beta, Delta and Omicron VOCs along the study in all SI-2 samples analyzed.

Week	Sample	Alpha	Beta	Delta	Omicron
1	1.1	6,36E+04	0,00E+00		
2	2.1	2,05E+05*	0,00E+00		
3	3.1	1,17E+06*	0,00E+00		
4	4.1	0,00E+00	0,00E+00		
5	5.1	4,86E+05	0,00E+00		
6	6.1	4,69E+04	0,00E+00		
7	7.1	5,21E+06*	1,63E+03		
8	8.1	3,03E+04	0,00E+00		
9	9.1	0,00E+00	0,00E+00		
10	10.1	2,10E+05	0,00E+00		
11	11.1	1,88E+04	0,00E+00		
12	12.1	1,62E+04	0,00E+00		
13	13.1	9,34E+03	0,00E+00		
14	14.1	4,48E+04	0,00E+00		
15	15.1	3,23E+04	1,20E+04*		
16	16.1	8,86E+04	0,00E+00		
17	17.1	2,47E+05	7,11E+05		
18	18.1	8,75E+05	0,00E+00	0,00E+00	
19	19.1	0,00E+00	0,00E+00	0,00E+00	
20	20.1	2,85E+03	0,00E+00	0,00E+00	
21	21.1	0,00E+00	0,00E+00	0,00E+00	
22	22.1	8,86E+03	0,00E+00	0,00E+00	
23	23.1	0,00E+00	0,00E+00	1,33E+05	
24	24.1	9,52E+03	0,00E+00	2,35E+05	
25	25.1	0,00E+00	0,00E+00	6,24E+03	
26	26.1	0,00E+00	0,00E+00	5,01E+03	
27	27.1	2,80E+05*	0,00E+00	2,18E+05*	
28	28.1	1,82E+05*	0,00E+00	1,60E+05	
29	29.1	0,00E+00	0,00E+00	6,71E+05	
30	30.1	0,00E+00	0,00E+00	3,60E+05*	
31	31.1	0,00E+00	0,00E+00	7,90E+05	

32	32.1	0,00E+00	0,00E+00	1,46E+05	
33	33.1	7,15E+03	0,00E+00	1,56E+05*	
34	34.1	0,00E+00	0,00E+00	1,12E+05*	
35	35.1	1,09E+05*	0,00E+00	1,93E+05	
36	36.1	0,00E+00	0,00E+00	2,30E+04*	
37	37.1	2,25E+03	0,00E+00	4,24E+03	
38	38.1	0,00E+00	0,00E+00	3,25E+04	
39	39.1	0,00E+00	0,00E+00	2,51E+03	
40	40.1	0,00E+00		3,42E+05*	
41	41.1	0,00E+00		6,39E+03	
42	42.1	0,00E+00		1,80E+04	
43	43.1	0,00E+00		0,00E+00	
44	44.1	0,00E+00		2,63E+03	
45	45.1	0,00E+00		2,27E+04	
46	46.1	0,00E+00		4,82E+06*	0,00E+00
47	47.1	0,00E+00		3,26E+05	0,00E+00
48	48.1	0,00E+00		4,76E+04	0,00E+00
49	49.1	0,00E+00		6,35E+04	0,00E+00
50	50.1	0,00E+00		2,14E+06*	2,38E+05*
51	51.1	0,00E+00		0,00E+00	5,17E+05
52	52.1	0,00E+00		1,50E+04	4,84E+06*

* inhibition

Supplementary Table S4. Temporal changes in the detection frequency (%) of Alpha, Beta, Delta and Omicron VOCs in wastewater from WWTP using RT-qPCR. Data are the frequency of positives for each VOC in all samples collected during each month.

Sampling date	Alpha	Beta	Delta	Omicron
January 2021	92.3	7.7		
February 2021	75.0	16.7		
March 2021	85.7	0.0		
April 2021	100.0	10.0		
May 2021	66.7	11.1	11.1	
June 2021	62.5	0.0	12.5	
July 2021	33.3	22.2	100.0	
August 2021	11.1	11.1	100.0	
September 2021	0.0	0.0	88.9	
October 2021	25.0	0.0	75.0	
November 2021	0.0		88.9	20.0
December 2021	0.0		77.8	88.8
January 2022	0.0		37.5	100.0

Supplementary Table S5. Temporal changes in the detection frequency (%) of Alpha, Beta, Delta and Omicron VOCs in wastewater from SI-1 using RT-qPCR. Data are the frequency of positives for each VOC in all samples collected during each month.

Sampling date	Alpha	Beta	Delta	Omicron
January 2021	100.0	25.0		
February 2021	100.0	25.0		
March 2021	100.0	0.0		
April 2021	75.0	0.0		
May 2021	50.0	0.0	0.0	
June 2021	75.0	0.0	25.0	
July 2021	60.0	40.0	100.0	
August 2021	25.0	25.0	100.0	
September 2021	0.0	0.0	100.0	
October 2021	0.0		75.0	
November 2021	0.0		75.0	0.0
December 2021	0.0		60.0	80.0

Supplementary Table S6. Temporal changes in the detection frequency (%) of Alpha, Beta, Delta and Omicron VOCs in wastewater from SI-2. Data are the frequency of positives for each VOC in all samples collected during each month.

Sampling date	Alpha	Beta	Delta	Omicron
January 2021	75.0	0.0		
February 2021	100.0	25.0		
March 2021	80.0	0.0		
April 2021	100.0	50.0		
May 2021	50.0	0.0	0.0	
June 2021	50.0	0.0	75.0	
July 2021	40.0	0.0	100.0	
August 2021	25.0	0.0	100.0	
September 2021	40.0	0.0	100.0	
October 2021	0.0		75.0	
November 2021	0.0		100.0	0.0
December 2021	0.0		80.0	60.0

Supplementary Table S7. VOC proportion (%) in the clinical samples analyzed.

Week	Alpha	Delta	Omicron
**3**	56.3		
**4**	64.6		
**5**	62.7		
**6**	84.0		
**7**	79.6		
**8**	80.0		
**9**	98.0		
**10**	100.0		
**11**	100.0		
**12**	100.0		
**13**	81.6		
**14**	70.8		
**15**	90.0		
**16**	95.8		
**17**	98.5		
**18**	98.4		
**19**	99.5		
**20**	98.1		
**21**	98.4		
**22**	88.2		
**23**	79.4		
**24**	74.4	7.7	
**25**	60.8	6.8	
**26**	44.3	46.6	
**27**	31.1	62.4	
**28**	29.9	63.8	
**29**	23.7	71.1	
**30**	21.4	78.1	
**31**	11.8	82.1	
**32**	6.5	92.1	
**33**	1.9	95.7	
**34**	0.4	99.1	
**35**	0.3	99.5	
**36**		100.0	
**37**		98.1	
**38**		100.0	
**39**		100.0	
**40**		94.4	
**41**		100.0	
**42**		95.3	
**43**		100.0	
**44**		97.2	
**45**		96.2	
**46**		98.2	
**47**		96.7	
**48**		97.5	
**49**		92.4	3.8
**50**		50.6	38.8
**51**		26.9	52.7
**52**		6.5	85.5
**53**		3.8	77.5
**54**		1.4	77.4
**55**		0.5	96.5
**56**		1.0	99.0
